# Supplementary material for: Particle-based hydrogel inks and support matrices for biofabricating structural complexity, soluble gradients, and cell-lined channels in fully granular bioprinted systems
Source: Biofabrication. Author manuscript; Available in PMC 2026 Aug 31. (PMC13527724; doi:10.1088/1758-5090/adfe97)
Supplement: Supplementary file 1 [file NIHMS2205210-supplement-Supplementary_material.pdf]

Particle-based hydrogel inks and support matrices for biofabricating structural complexity, soluble gradients, and cell-lined channels in fully granular bioprinted systems

Julia Tumbic, Emily Ferrarese, Remington Martinez, Thomas Ackleson, Daniel Delgado and Christopher B Highley

Supplementary data

SI Table 1: Average particle size and CV

Material	Diameter (μm)	CV (%)	n
NorHA-8	9.63 ± 4.50	46.7	393
NorHA-4	16.44 ± 10.47	63.7	466
NorHA-2	16.45 ± 9.81	59.6	427
Gelatin Ink	4.60 ± 3.77	82.0	520

SI Table 2: Herschel-Bulkley parameters

Material	Yield Stress (Pa)	K	n
NorHA-8	50.40 ± 17.47	0.90 ± 0.57	0.81 ± 0.10
NorHA-4	16.97 ± 2.71	1.52 ± 0.72	0.69 ± 0.03
NorHA-2	5.97 ± 1.32	0.63 ± 0.30	0.74 ± 0.03
Gelatin Ink	72.73 ± 11.44	11.83 ± 8.95	0.56 ± 0.11

SI Table 3: Strain sweep onset and G’-G” crossover

Material	Onset (%)	G’-G” Crossover (%)
NorHA-8	18.94 ± 7.27	37.96 ± 12.13
NorHA-4	26.80 ± 1.61	54.27 ± 5.83
NorHA-2	51.96 ± 20.73	124.16 ± 11.37
Gelatin Ink	24.55 ± 10.76	62.53 ± 30.23

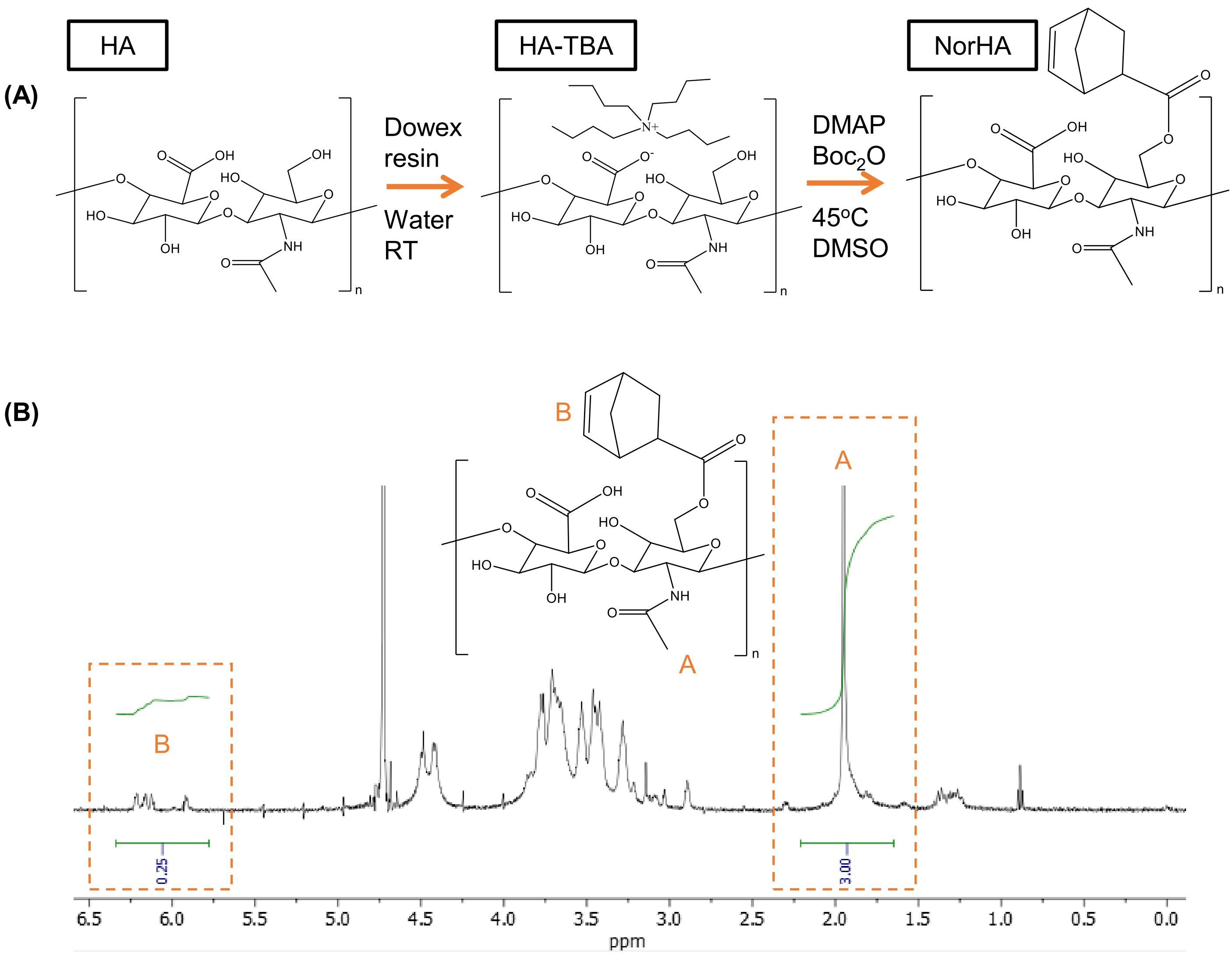

SI Fig. 1: (A) synthesis of NorHA using a multistep process, and (B) an example of NMR spectrum for NorHA, showing roughly 12.5% modification.

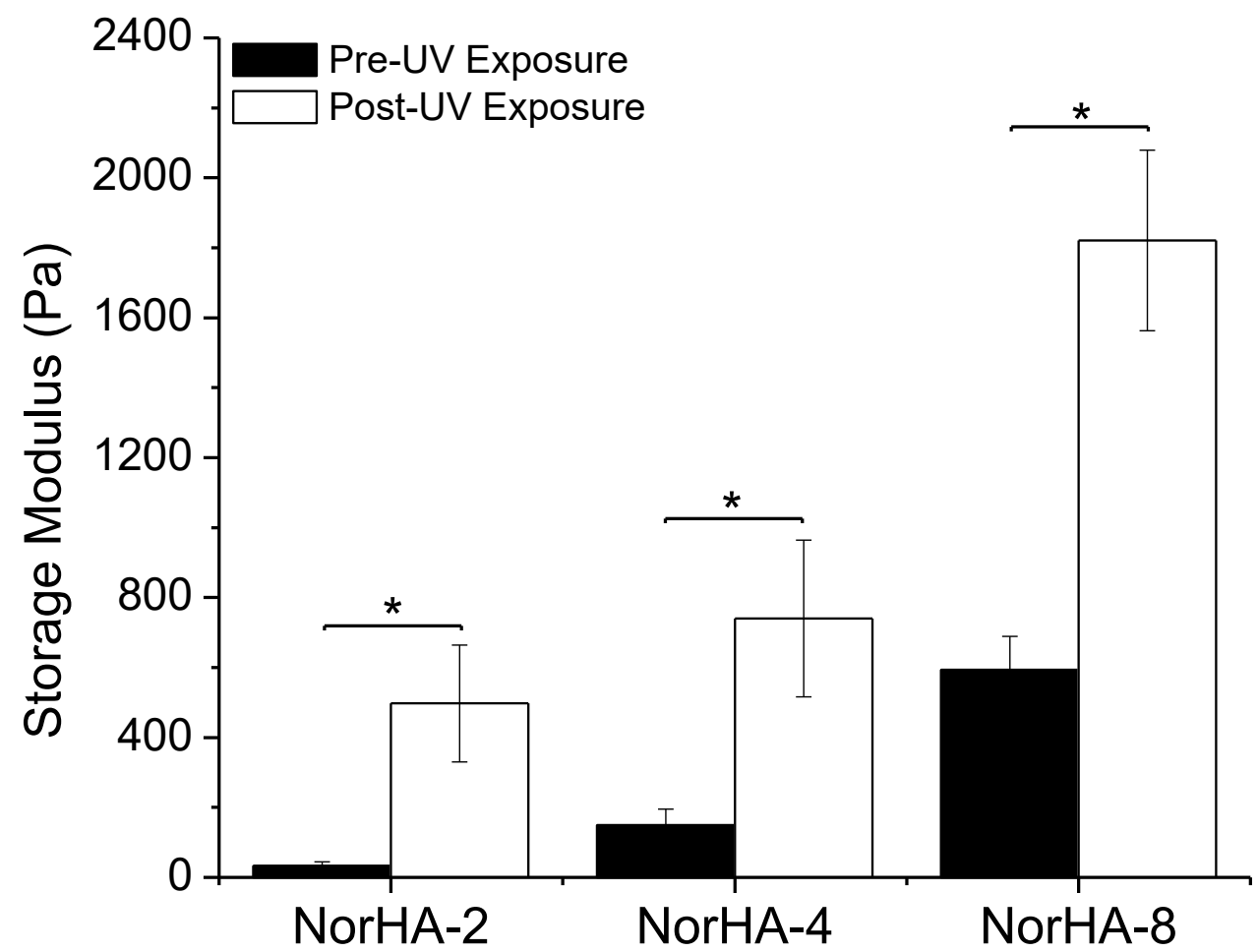

SI Fig. 2: Average storage modulus before and after crosslinking of NorHA-2, -4, and -8 microgels with interstitial NorHA. As expected, the storage modulus is significantly different post-crosslinking, and is dependent on crosslinking density of the particles themselves.

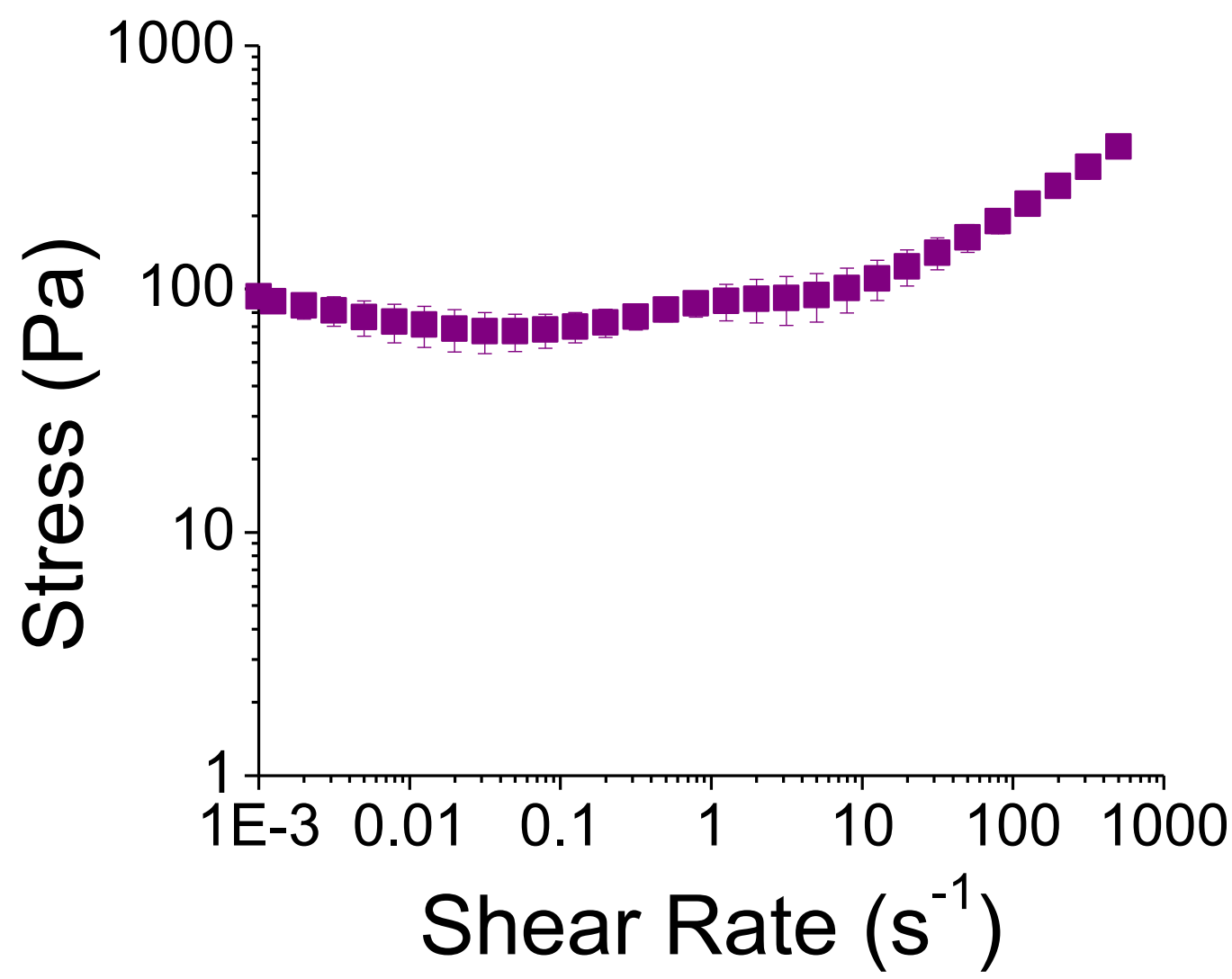

SI Fig. 3: Yield stress testing of gelatin microgel-based ink, with error bars denoting standard deviation.

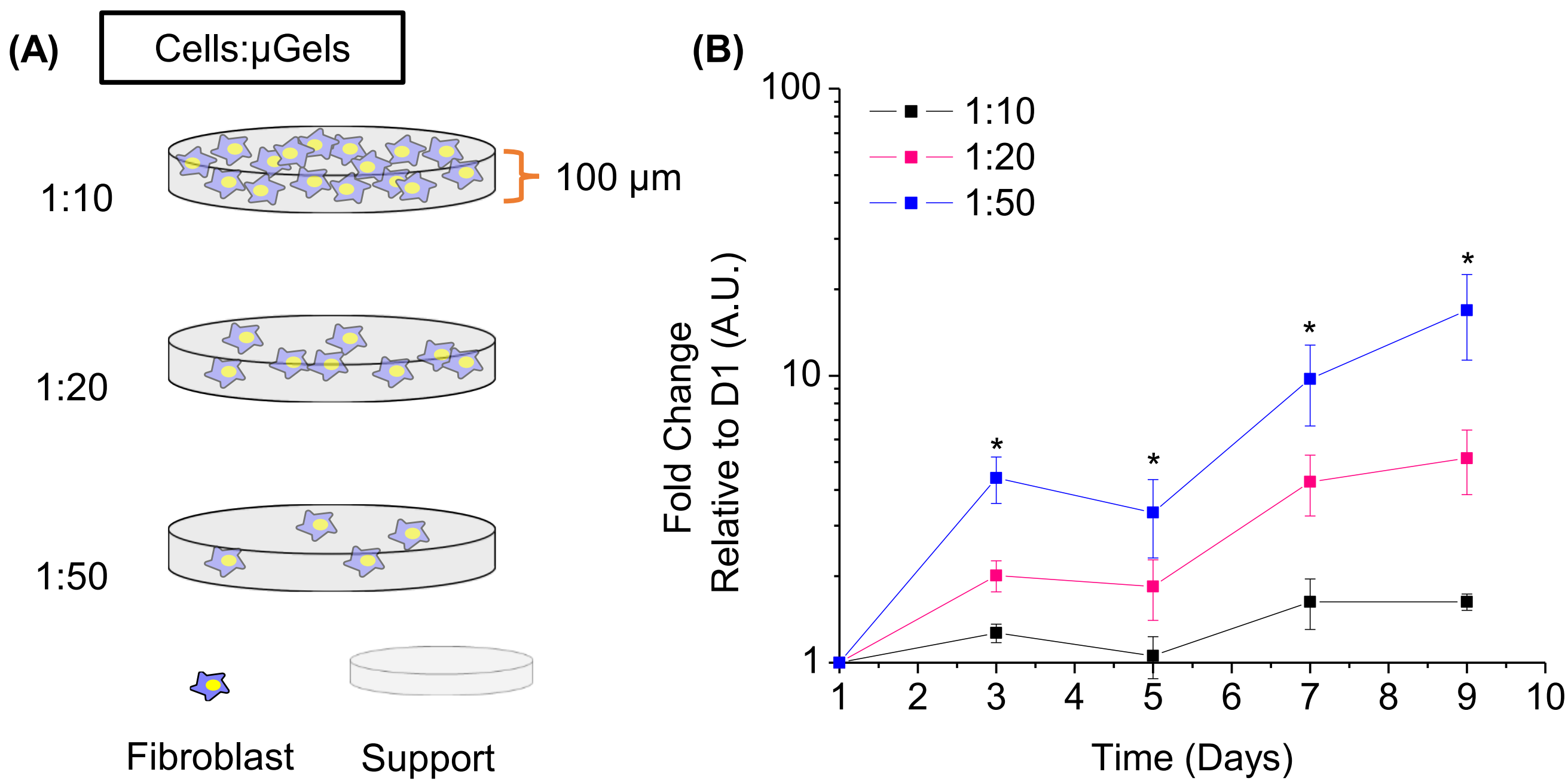

SI Fig. 4: Cell proliferation in thin gels as a function of cells density within support volume. (A) Schematic illustration of fibroblast proliferation experimental setup with varying cell densities within NorHA-4 supports, and (B) Alamar blue assay results over a 9-day time period showing either maintenance of cell viability or proliferation of fibroblasts within the NorHA-4 supports.

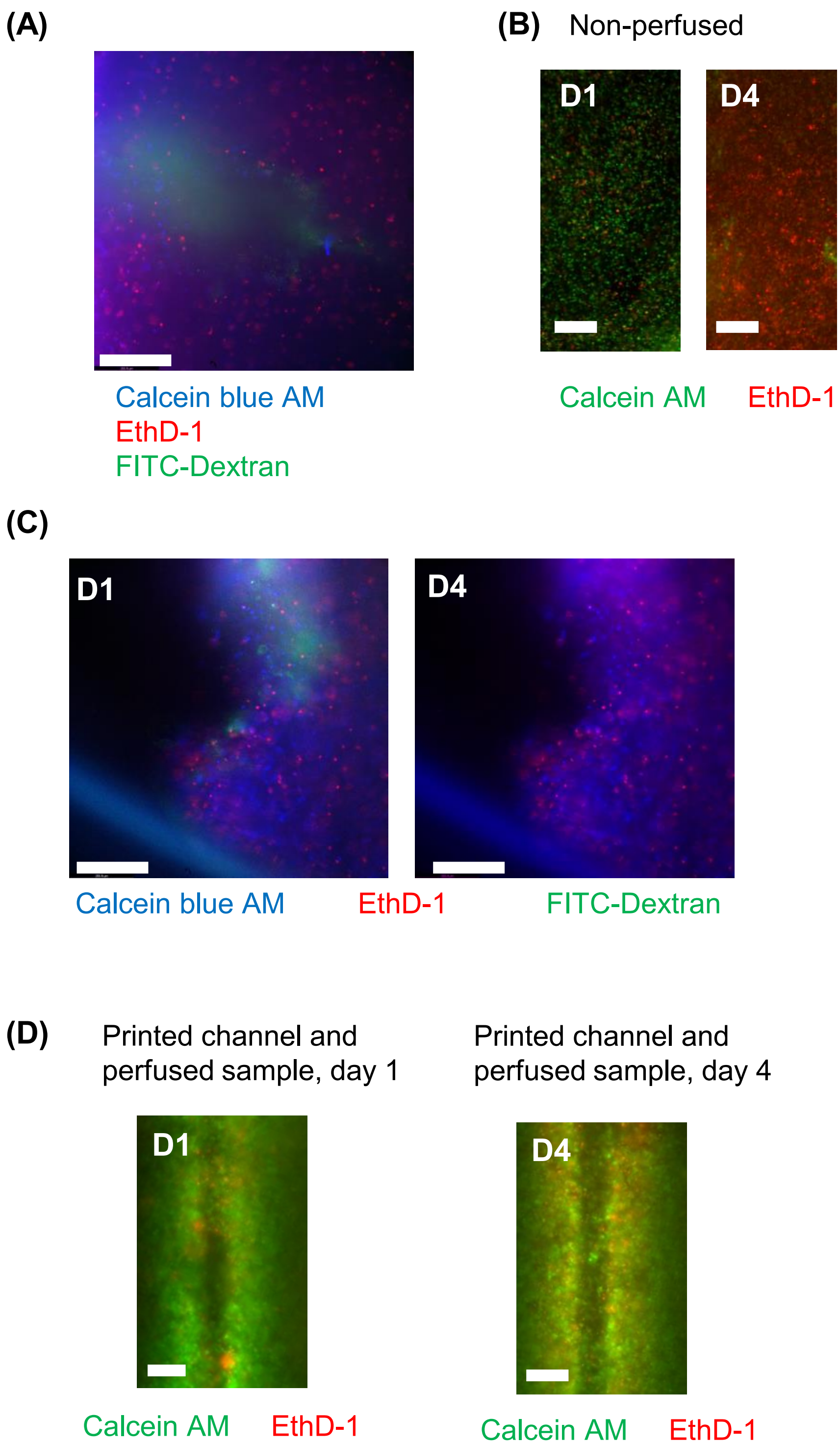

SI Fig. 5: Cell viability in granular supports is enhanced by the presences of channels. (A) Channel from Fig. 7 with FITC introduced into the channel lumen. (B) A non-perfused sample illustrating low viability from D1 to D4 as expected, (C) Additional samples with cross-sectioned channels on D1 and D4 showing enhanced viability at D4. (D) A longitudinal plane of channel on D1 and D4 showing enhanced viability around the channel on D4. Scalebars for all images are 300µm.

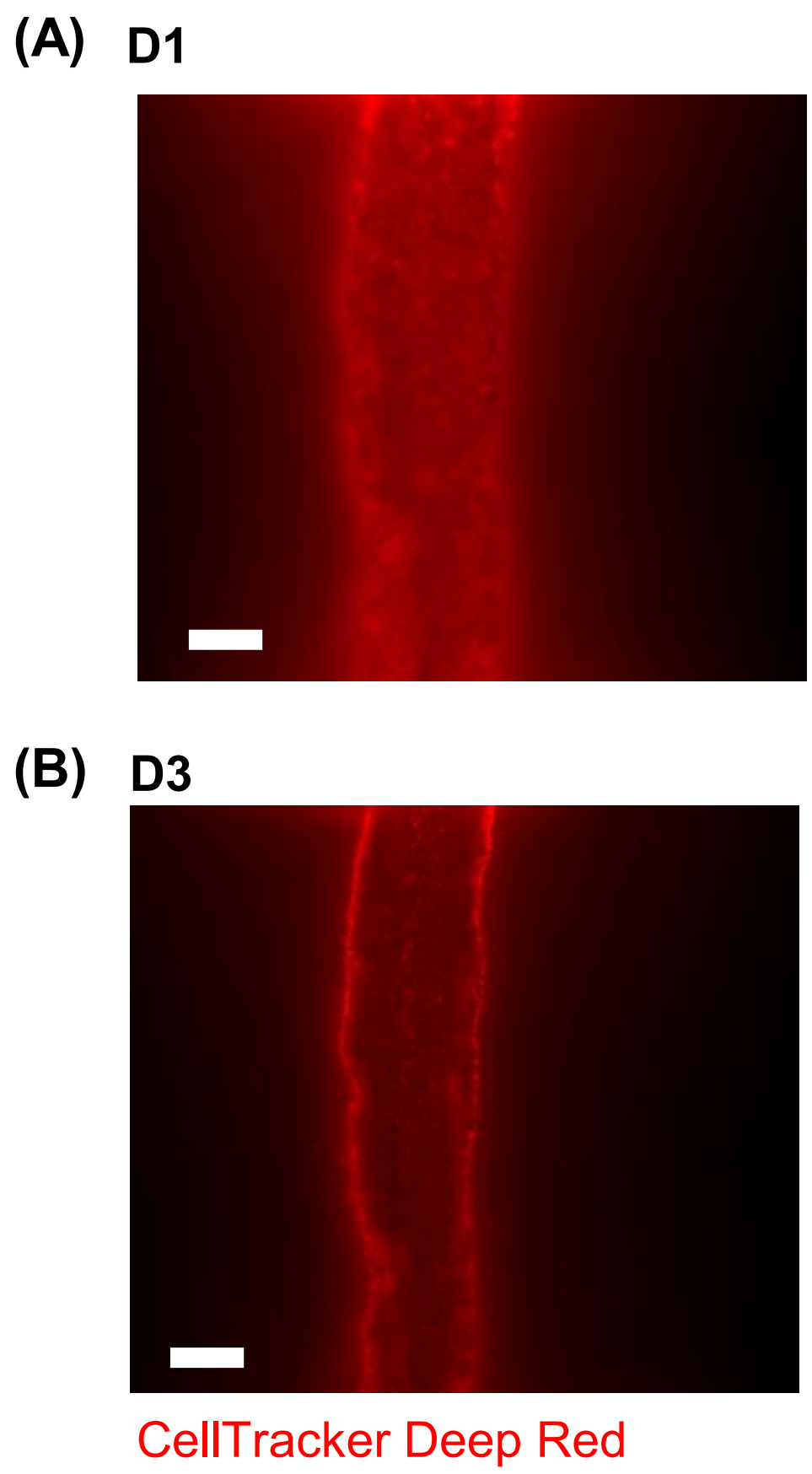

SI Fig. 6: Images of channel lined with HUVECs on (A) D1 post-seeding, and (B) D3 post-seeding. Scalebars: 300 μm.

(A)

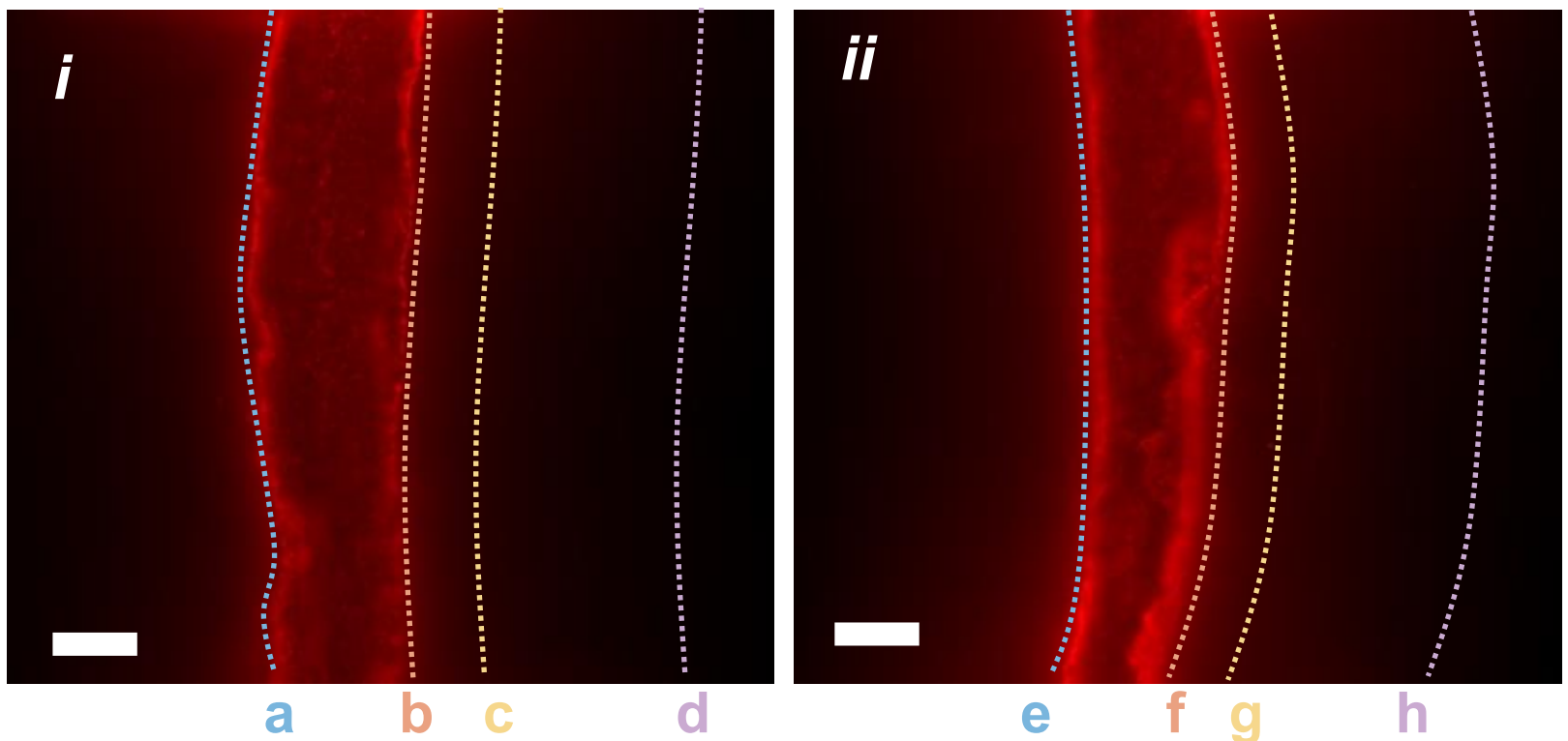

(B)

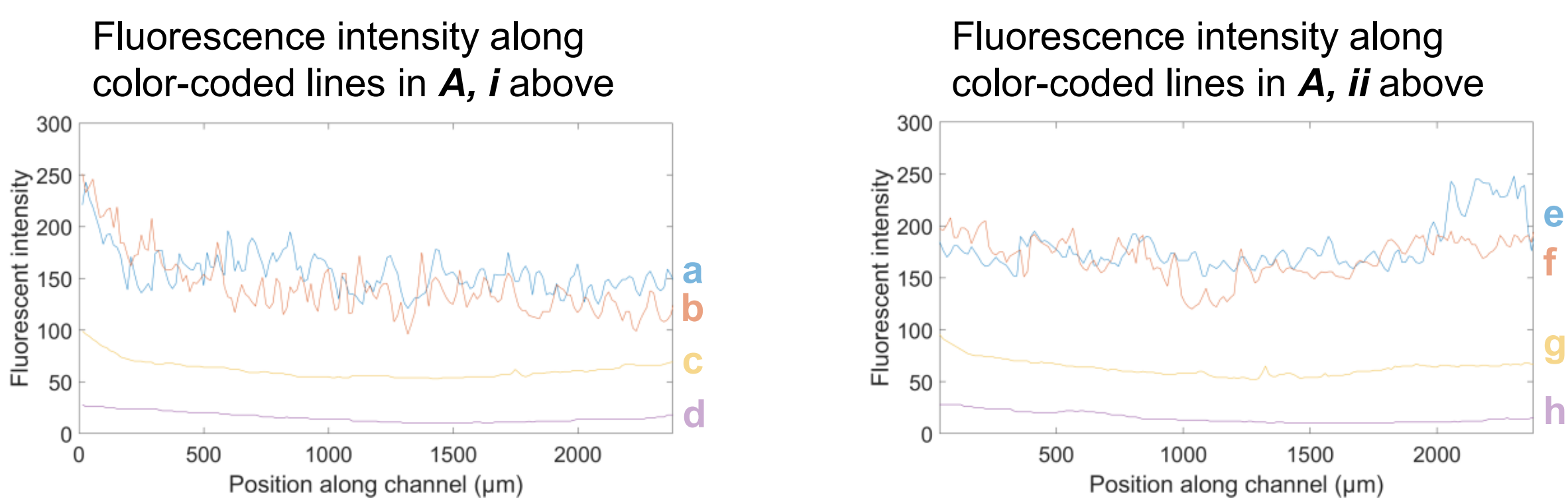

(C)

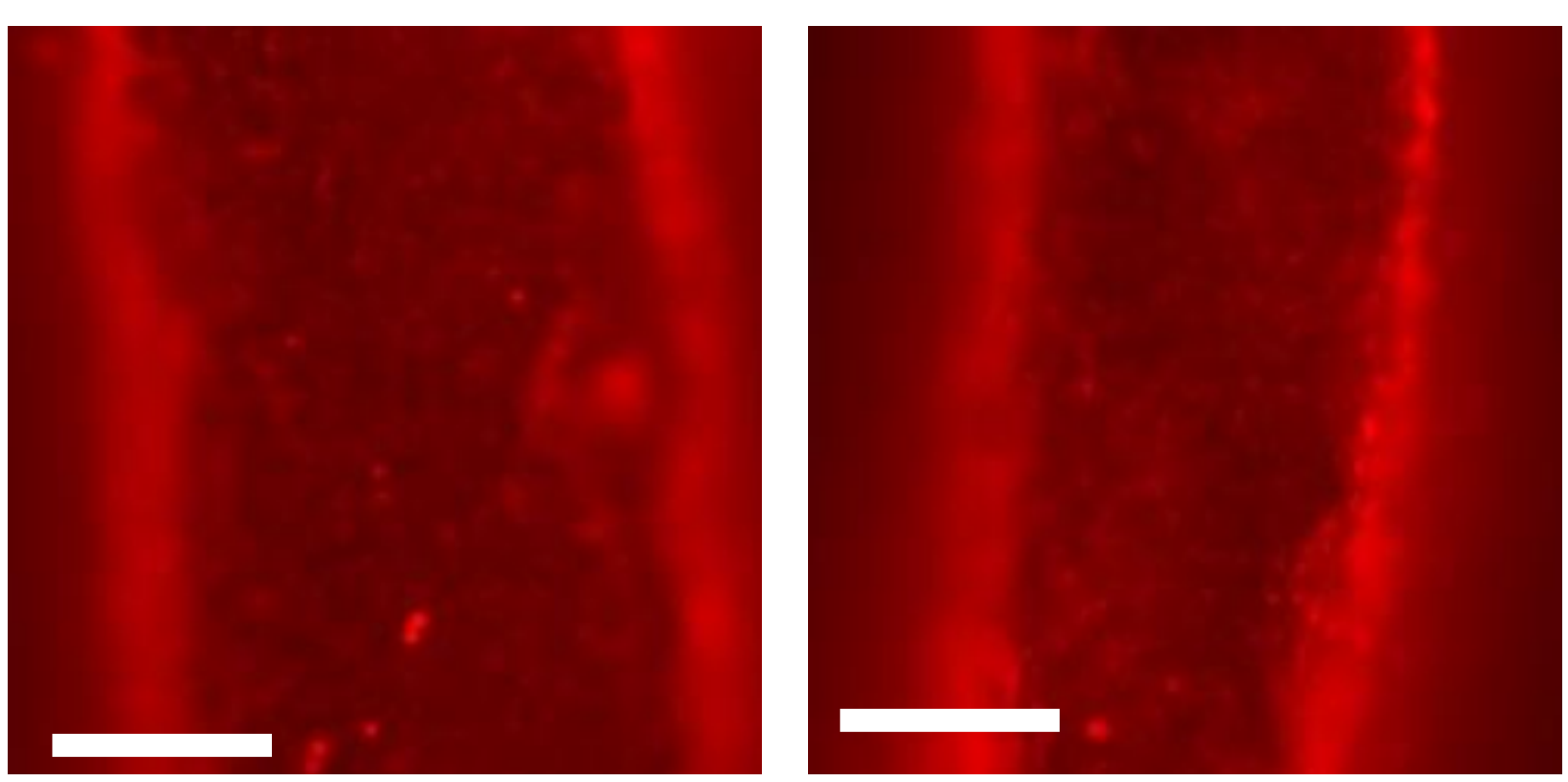

SI Fig. 7: (A) Images of channels lined with HUVECs on D3 post-seeding. Straight walls, indicated by arrows, used in analysis of coverage. (B) As a measure of cell coverage of channel walls, fluorescent intensity (minimum = 0, maximum = 255) is quantified using MATLAB's improfile function. HUVECs are labeled with CellTracker dye. Intensities are quantified along the dotted lines (indicated by color code and letter labels), with quantification going from top to bottom along the lengths of ~2400 µm channels. (C) Images from focal planes near top surfaces of channels showing HUVEC coverage across channel width.

Scalebars: 300 µm (A) and 150 µm (B).

(A) (B)

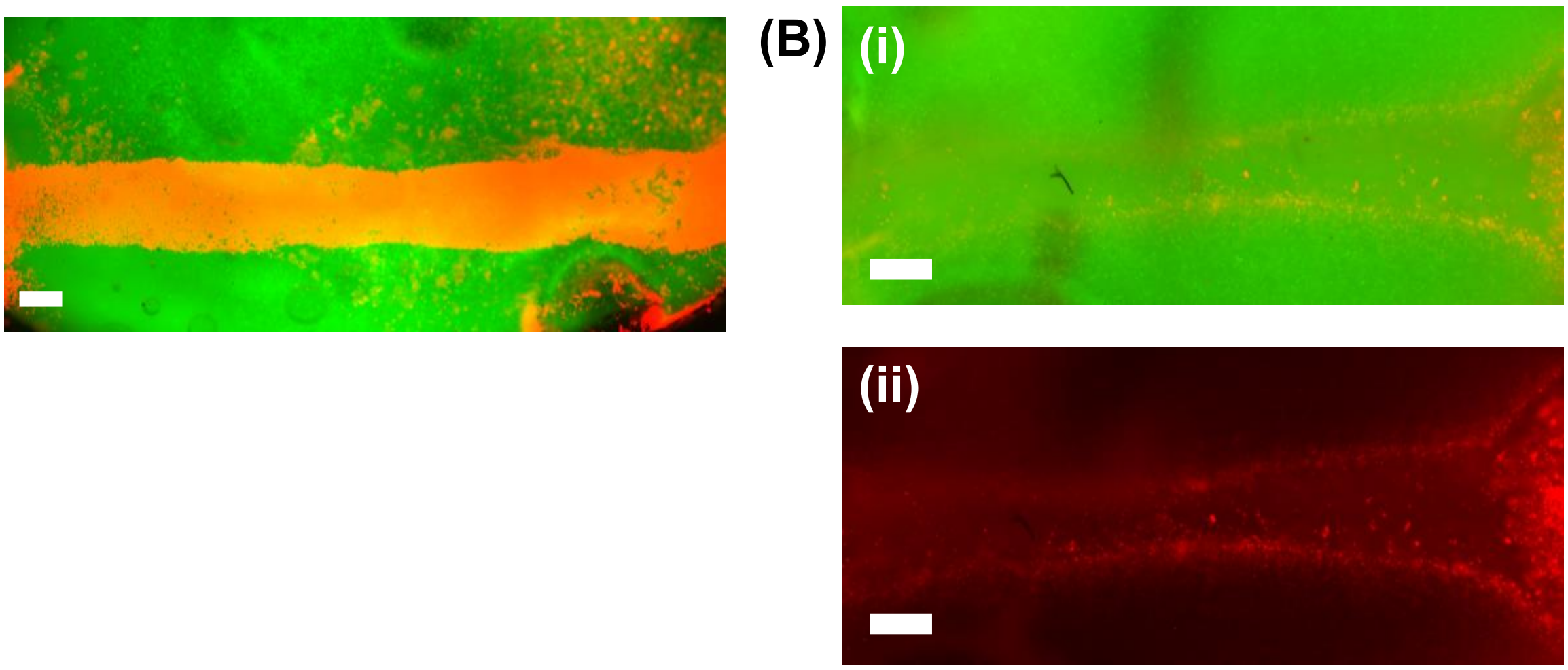

SI Fig 8: Towards endothelializing channels that are printed within granular supports that include dense cell populations: A) a channel printed into a NorHA support containing MSCs (green CellTracker dye) has been filled with HUVECs (red CellTracker dye) to seed an endothelial cell layer along the channel walls. B) After washing unadhered HUVECs from the channel in *A)* and incubating for 2 hours, HUVECs are observed adhering to the channel walls. i) Image with the HUVECs' (red) and MSCs' (green) fluorescent signals overlaid. ii) Image of HUVECs only show cell-lined channel walls at 2 hours post-HUVEC seeding. Scalebars: 200 μm.
